# A simulation model to predict the most efficient way to utilise operational resources when vaccinating badgers against bTB

**DOI:** 10.1371/journal.pone.0354329

**Published:** 2026-07-27

**Authors:** Richard Budgey, Megan Bird

**Affiliations:** 1 National Wildlife Management Centre, WOAH Collaborating Centre in Risk Analysis and Modelling, Animal and Plant Health Agency, Sand Hutton, York, United Kingdom; 2 National Wildlife Management Centre, Animal and Plant Health Agency, Woodchester Park, Gloucestershire, United Kingdom; The University of Georgia, UNITED STATES OF AMERICA

## Abstract

Bovine tuberculosis (bTB) is a disease carried by badgers that seriously impacts the health and productivity of cattle in the United Kingdom (UK). The UK government’s aim is to be officially TB-free in cattle by 2038 in England and badger vaccination is one approach that can be used to achieve this. We use a simulation model to compare the relative performance of different vaccination strategies (treatment every year, every second year and every third year) under different field conditions and assumptions about vaccine efficacy. Population density, disease prevalence and duration of vaccine-induced immunity substantially affected the outcome of vaccination. A vaccine strategy with lower frequency treatment was effective and offers operational efficiency as multiple areas can be treated near concurrently by a single vaccination team, potentially halving the cost per unit area. The information presented here may be used to guide operational managers and decision makers towards a more efficient strategy in a particular area and may provide an estimation of the likely success of the chosen strategy.

## Introduction

In parts of the United Kingdom, badgers are a sylvatic host of bTB and are considered to form a bi-directional disease system with cattle [1,2]. The reduction, or even elimination, of bTB in badgers is therefore an important part of the national bTB strategy to achieve Official TB-Free status [3]. Different approaches have been tried to achieve this aim in the last several decades including reactive (following detection in cattle) and proactive (whole area) badger culling and vaccination [4].

Culling has the benefit of rapidly reducing the number of infectious animals within the culled area, so newly added susceptible individuals have a reduced likelihood of becoming infected once control ends. However, it may lead to a perturbation effect, whereby the removal of animals from a population causes an increase in movement, potentially increasing disease transmission [5]. Vaccination can prevent susceptible animals from becoming infected and reduces disease prevalence over time without affecting social structure, thereby avoiding the risk of perturbation. It is considered more acceptable to the public and is considered most effective if used to maintain low levels of disease already achieved by culling [6].

In 2013, a policy of proactive badger culling over large (100–300 km^2^) areas was initially adopted in the High Risk Area (HRA) of England and subsequently extended to include the Edge Area (boundary of the high and low cattle TB risk areas of England). In addition, ad-hoc vaccination was undertaken where there was sufficient local interest and resource. In 2024, the policy of extensive culling was ended, with the intention of increasing the area of under vaccination [7].

Those responsible for directing and targeting the operational management of disease in badger populations will wish to have some estimate of the likely success of vaccination. Vaccination effectiveness is dependent on a number of factors, some of which, such as badger population density and disease prevalence are specific to local areas and may be estimated in the field. Other factors, including the efficiency of field operations and the efficacy and mode of operation of vaccines, may be unknown. Given the level of uncertainty involved, modelling can provide an estimate of vaccination efficacy for the range of field and vaccine conditions that could be encountered by operational managers and thus help inform how badger vaccination is deployed and targeted in the field.

The vaccine used on badgers in the UK (BadgerBCG [8,9]) is given by intra-muscular injection, requiring animals to be trapped and restrained. A large trapping campaign takes substantial resource, requiring a skilled team with good local knowledge [10,11], but even then, trapping efficiency may be limited by incomplete access to land, difficult habitat in which to find setts and place traps, and necessary consideration of potential regulatory requirements. Capture efficacy of between 50% and 70% of a population trapped each year are estimated in practice [10–12].

No robust research on the duration of BCG vaccine-induced protective immunity (DoI) in badgers has been undertaken. The average lifespan of a badger is about three to four years and few live as long as 10 years [13–15], so if DoI is no more than one or two years, the protection given to a vaccinated badger population will soon be compromised.

We modelled different vaccination strategies to assess their effectiveness under a range of conditions and to help managers deploy resources where they would likely achieve the greatest overall benefit. We also aim to show when benefit may be gained by reducing the frequency of vaccination from annually to every second or every third year. Potential operational efficiencies are thereby available as two or three areas can be treated by a single field team in alternating years, so the delayed start of several years that would be necessary from treating sites sequentially can be avoided.

## Methods

TBi is an individual-based, stochastic computer simulation model, written in the Python 3 programming language [16], that simulates the behaviour of individual badgers, parameterised to produce a stable population and disease epidemiology using a two-month timestep [17–19]. The potential effect of population and disease management approaches can then be assessed. The model and motivation for development are described elsewhere, including a sensitivity analysis [20–22]. While validation of wildlife disease intervention models is challenging due to the lack of data, the model has recently been successfully validated against Test, Vaccinate or Remove (TVR) pilot trial in Northern Ireland [23], which suggests it can be used to robustly predict the effect of vaccination at a population level. All model parameters and their source and a full description of the model using the ODD protocol [24,25] is in supplementary S1 File.

The model arena comprises 100 x 100 grid cells, wrapped to form a torus to eliminate edge effects across which 300 badger social groups are randomly but homogeneously distributed at a mean density of 0.75 km^-2^. Grid cells nominally have an area of 0.04 km^2^ however population and disease processes occur only within a network of neighbouring social groups and thus area and distance are disregarded after initial arena generation. A similar number of farms (215) are also randomly distributed across the arena and used to determine participation in management; each farm is given a 70% probability of participation based on experience of prior management operations [26,27]. Badger management occurs with a central circular core of 100 km^2^ containing 85 badger social groups, but the probability that individual groups undergo management is determined by the participation status of overlapping farms. All badgers within participating land in the central core were vaccinated in June of each year if successfully trapped and a probability of 50% for each badger being trapped each year was assumed. Outside the central core is a larger, non-controlled area. Both core and outer area have the same population density and background disease prevalence at the start of the management period.

### Characterisation of individual badgers

Population dynamics parameters are taken from the literature and from the population at the Animal and Plant Health Agency’s (APHA) Woodchester Park research station in Gloucestershire, UK. Individual badgers are characterised by the state variables social group, sex and age, health status and immunity status. Age categories are cub, yearling (one-year old), and adult. Health status categories are healthy, infected, single-site and multi-site excretor (*Mycobacterium bovis* isolated from multiple body sites, e.g., sputum, faeces, urine or bite wounds) [28] and probability of disease progression based on field data from Woodchester Park [28]. Immunity states follow three vaccine pathways: no protection (same probability of becoming infectious as an unvaccinated animal, applied to 20% of animals), partial protection (probability of becoming infectious half that of unvaccinated animal, applied to 10% of animals) and full protection (zero probability of infection, applied to 70% of animals) based on an expert review of BCG experiments in badgers [29]. Probabilities are set independently for each animal. Further vaccination while still immune has no effect whereas re-vaccinating once immunity is lost results in additional protection, with the same probability of full or partial protection as at initial vaccination.

Reproduction is simulated as a density dependent process, based on a maximum number of litters for each social group. Litter size is determined probabilistically from a distribution of known litter sizes [30], with a mean of 2.94 cubs per litter, and a sex ratio of 1:1. Mortality rates are sex and age-related, based on data from Woodchester Park [28] and badgers in their first two months of life – those still underground – have a higher mortality rate than older animals [14]. Mortality is also elevated for animals in the bTB excretor disease classes [28].

Permanent dispersal to any other social group occurs with equal probability for all age classes throughout the year, with a higher probability of moving to a smaller group and higher probability of male dispersal. Badgers also move permanently to neighbouring social groups in response to demographic imbalance (i.e., excess males or females will disperse to a neighbouring territory where there are no individuals of that sex).

Disease transmission occurs between animals of the same social group and neighbouring groups; within-group transmission has a greater probability (20-fold) than between-group transmission to reflect the higher level of interaction expected within a group [31]. Transmission probability is increased as animals moved from bTB single-site excretor to bTB multi-site excretor class.

### Simulation experiment

The model was used to estimate the effectiveness of badger vaccination as a disease management tool under a variety of environmental and operational conditions. These comprised population density (mean social group size), equilibrium disease prevalence, duration of vaccine-induced immunity and capture probability and each combination of conditions was subject to alternative vaccination strategies. The model estimated population size, number of infected animals and number that were bTB immune. The various conditions were selected to be generally representative of those found in potential large-scale or smaller vaccination areas in the UK allowing any given area to be simulated by selecting the relevant combination of conditions.

### Parameters chosen for scenarios

In this study, the mean social group carrying capacity was adjusted to enable the production of populations with stable high (mean social group size 6.3, referred to as POP HI throughout the text) or low (mean social group size 4.5, POP LO) densities, representative of populations from the HRA and LRA [14,32]. In addition, a post-cull population (mean social group size 2.5) that returned to pre-cull density (mean social group size 6.3, POP POST-CULL) over the course of approximately ten years was included.

The probability of bTB transmission between individual badgers was adjusted so the stable population disease prevalence matched one of two values (0.2, referred to as PREV HI throughout the text and 0.05 PREV LO) representative of populations in the HRA and LRA.

In the absence of data on duration of BCG vaccine-induced immunity in badgers, four alternatives have been included to ensure the true duration of immunity was captured. The duration of vaccine-induced immunity was one, two or three years, after which badgers lost protection, or for the lifetime of the animal. These are referred to as DOI1, DOI2, DOI3 and DOI LIFE.

### Simulation of management operations

Badgers were vaccinated according to one of three alternative vaccination strategies: vaccination every year, every other year and every third year. For a fair comparison, annual vaccination was undertaken for five years (referred to as V11), every other year for ten years (V12) and every third year for fifteen years (V13); strategies therefore received equal operational resourcing. By duplicating output from a single area, it was possible to compare the effectiveness of concurrent vaccination in multiple areas with similar population density and disease prevalence using V12 or V13 with sequential vaccination in multiple areas using V11.

All badgers within participating land in the central core were vaccinated in June of each year if successfully trapped. An animal’s probability of capture was determined by one of three alternative trapping rates: 0.7 (referred to as TRAP HI), 0.5 (TRAP MED) and 0.3 (TRAP LO), which reflect both the level of available operational resource and the effect of local conditions. They are representative of trapping rates seen in the field [27], but are considered to range from optimistic to pessimistic.

### Simulations

Simulations included a burn-in period of 100 years to allow populations and disease to stabilise. Vaccination started in year 101 and continues for five (V11), ten (V12) or 15 (V13) years. For POP POST CULL scenarios, a POP HI population was reduced by approximately 50% each year between years 97 and 100 to produce a mean group size of 2.5 animals prior to vaccination.

## Results

Model output was produced once per year immediately following management activity. While the badger population would have changed throughout the year due to reproduction and mortality, annual output has sufficient temporal resolution to demonstrate the effect of different management strategies. Model output was available for the core area comprising population size, number of infected badgers (Elisa positive, single- and multi-site excretors) and hence disease prevalence, number of trapped badgers, and the number with immunity at one time. All explored strategies are included: V11, V12, V13 and the do-nothing strategy, business as usual (BAU). As the badger population had stabilised by year 100, no further change in disease levels would be expected under BAU and variation seen in this output is due to stochastic variation arising from the random selection of values from parameter distributions in the model. A subset of all possible conditions is presented in this text. TRAP HI, TRAP LO, PREV MED, DOI2 and DO LIFE have been omitted, as these outputs can be inferred from the data presented. The effect of variation across the full range of each condition is presented in supplementary information S2 File.

**Fig 1:**
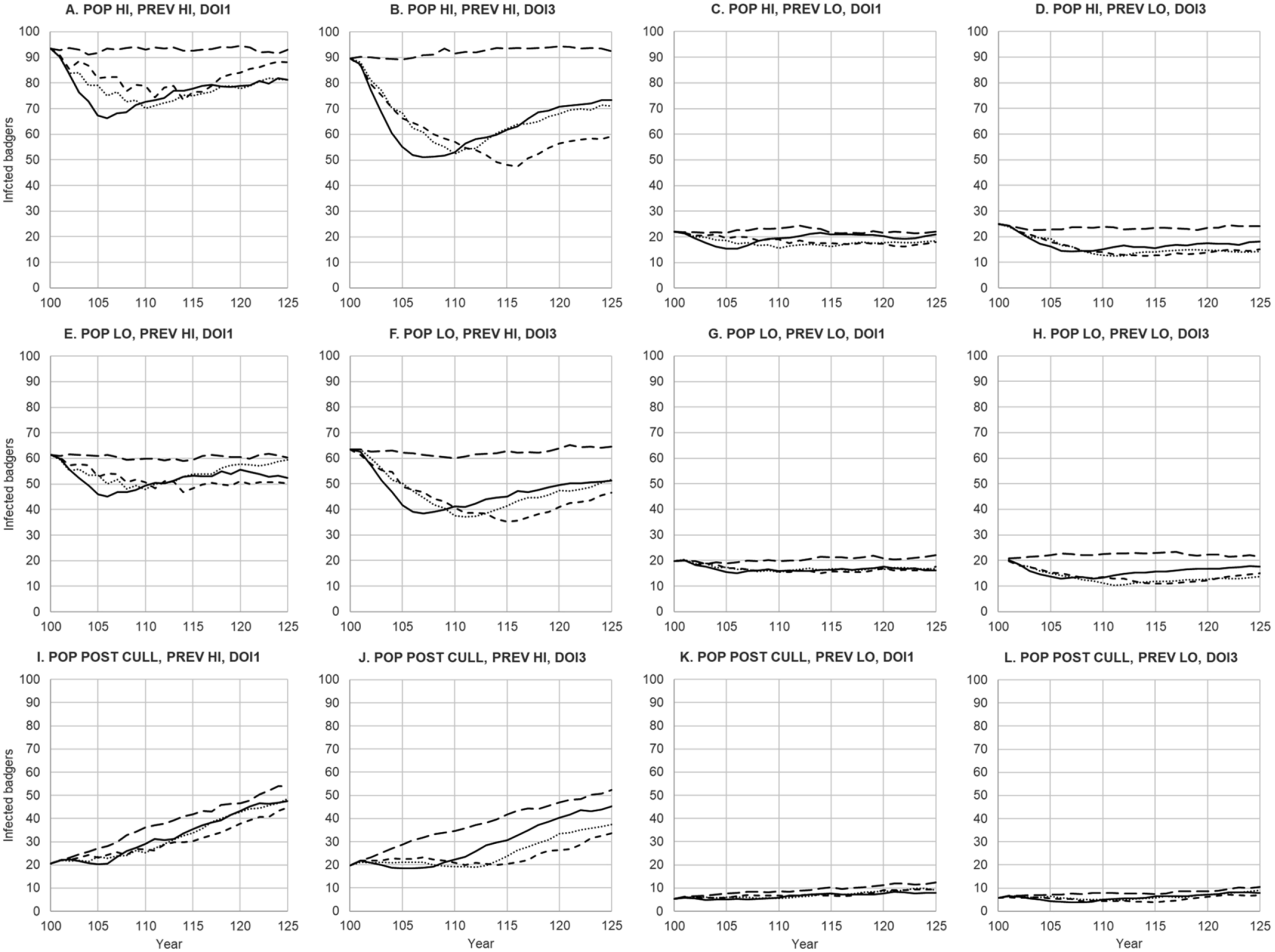
Number of infected badgers in core controlled area. V11 solid line, V12 dotted line, V13 dashed line, BAU long dashed line.

Under POP HI and POP LO, vaccination reduced the number of infected badgers in the population during the period of vaccine application and subsequently, during the duration of immunity (Fig 1). After protection ended, infection increased again but remained lower than before treatment for the remainder of the 25-year simulation period. A similar percentage reduction in the number of infected badgers was seen under all combinations of population density and prevalence, (between 22% and 30% with DOI1 and between 45% and 71% with DOI3, depending upon strategy). The greatest reduction in the number of infected badgers was achieved when population density and background prevalence were both high, irrespective of DoI or choice of vaccination strategy (Figs 1A and 1B). Under POP POST CULL, the reduction in infection levels achieved by culling were maintained during treatment, but no additional reduction was seen; infection levels then rose more rapidly than under POP HI or POP LO, but there were always fewer infected animals than under BAU (Figs 1I-L).

### Prevented infection

**Fig 2:**
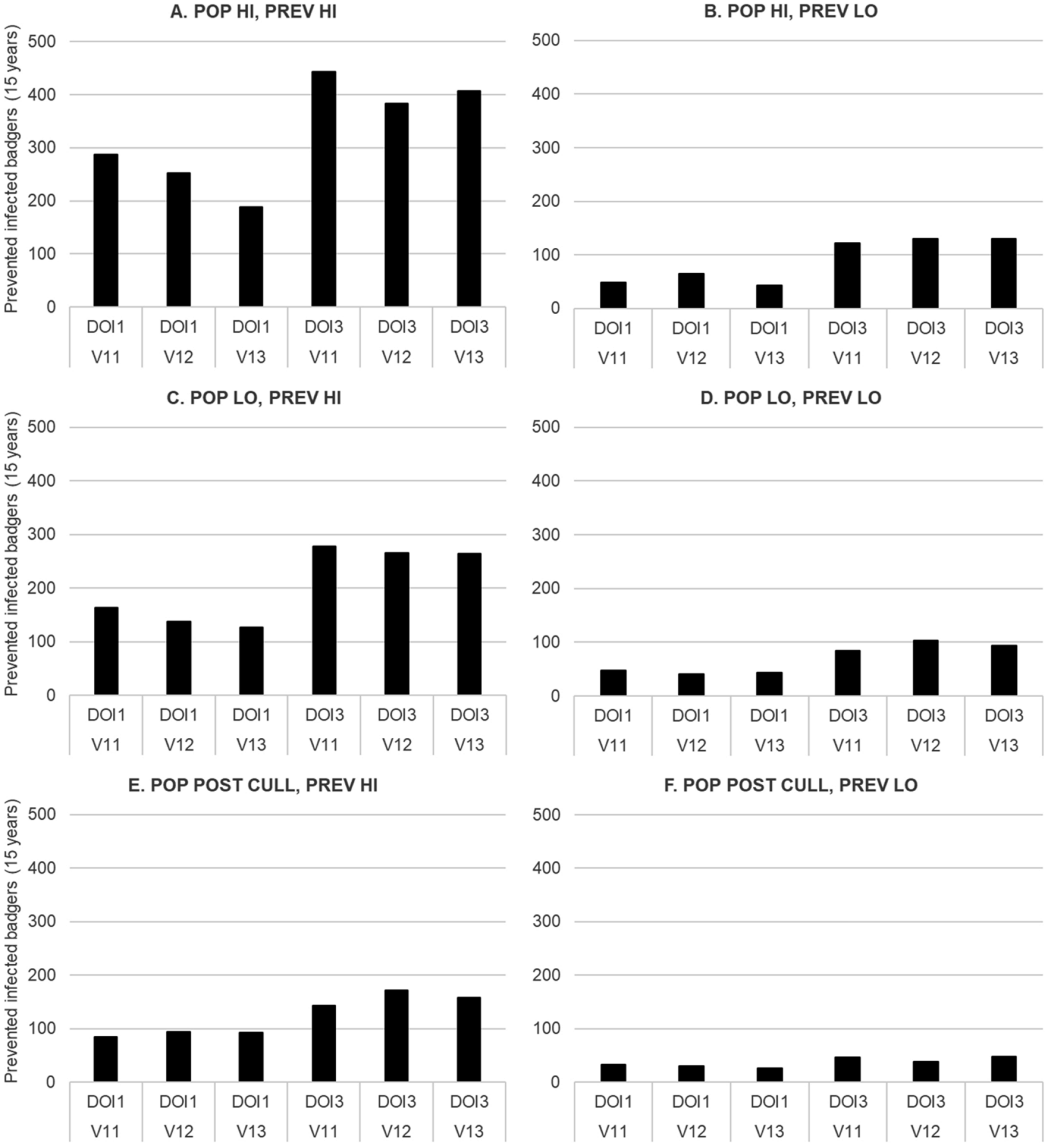
Prevented infected badgers over 15 years.

The number of prevented infected badgers is the difference between the number of infected badgers under the treatment compared to the number expected under BAU, summed over 15 years (Fig 2). Greater benefit was seen with higher population density and higher prevalence. The total number of infected badgers prevented over 15 years was highest with the combination POP HI and PREV HI (between 189 and 444 depending upon DoI and strategy; Fig 2A), and lowest with the combination POP POST CULL and PREV LO (between 26 and 48; Fig 2F).

### Effect of DoI

**Fig 3:**
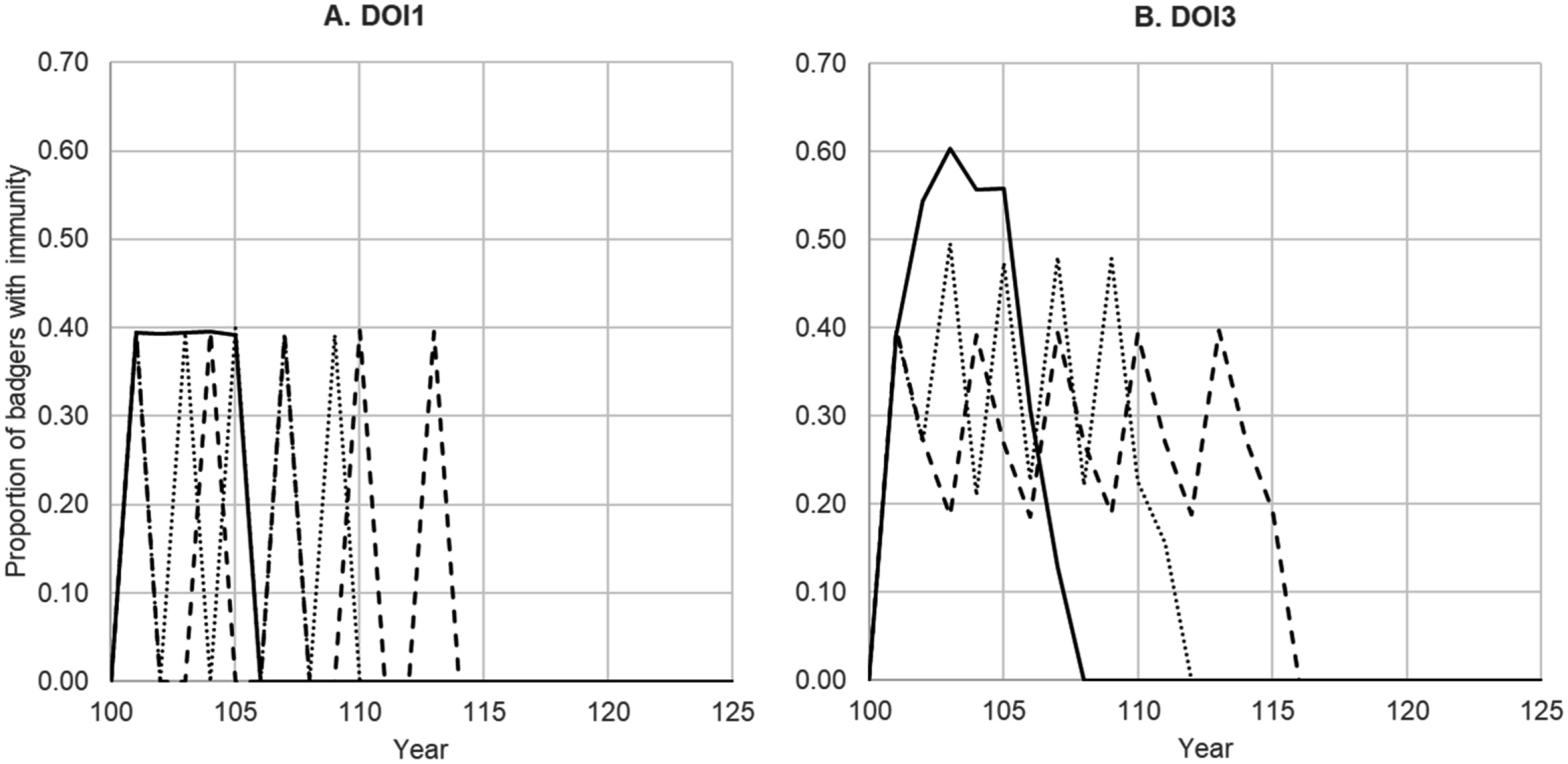
Proportion of badgers with immunity. V11 solid line, V12 dotted line, V13 dashed line. Proportions were similar for all combinations of population density and background prevalence. For DoIs longer than one year, it includes animals that have just received vaccine and those that were vaccinated in previous years.

Longer DoI resulted in improved performance for less frequent strategies, with DOI3 resulting in fewer infected badgers present at the end of the treatment period when culling had not occurred (e.g., Figs 3A and 3B). However, V11 was always the best strategy irrespective of DoI in terms of the total number of prevented infected badgers (e.g., Figs 2A and 2C). The effectiveness of vaccination is determined by the proportion of badgers with immunity and Fig 3 shows how vaccination strategy and DoI affect this proportion. When DoI was one year, continuous protection was only provided by V11; V12 and V13 resulted in years when no badgers had immunity. When DoI was three years, a proportion of badgers always had immunity under all strategies, and all strategies achieved similar success in terms of infected badgers prevented. DOI3 resulted in 30% to 50% extra badgers prevented compared to DOI1, irrespective of population density, background prevalence or strategy (Fig 2). Again, the greatest total number of infected badgers prevented was seen with PREV HI, particularly in combination with POP HI (Fig 2A).

### Effect of trapping efficacy

Trapping efficacy has a direct effect on the success of management. Higher trapping efficacy logically resulted in more animals with immunity. TRAP HI (70%) trapped 2.33 times more animals than TRAP LO (30%).

### Choice of strategy

In general, due to the frequency of application, V11 logically resulted in faster reduction of infection than V12 or V13. Conversely, due to the duration of each strategy, infection rose again soonest with V11, and latest with V13. This pattern was seen most clearly when both population density and disease prevalence were high, as the effect of vaccination was greater under these conditions (Figs 1A &B). While more frequent vaccination resulted in quicker reduction in infected badgers, the optimal strategy in terms of total infected badgers prevented over 15 years was dependent upon other factors. Under PREV HI and DOI1 in combination with POP HI or POP LO, more frequent vaccination strategies performed better, but under other combinations, no strategy was clearly optimal. (Fig 2).

Near-concurrent treatment of multiple areas is possible under strategies V12 and V13, as one area can be treated during the ‘off’ year of another by a single field team. The alternative, under V11, is sequential treatment, when five years of vaccination are completed in one area before another is started.

**Fig 4:**
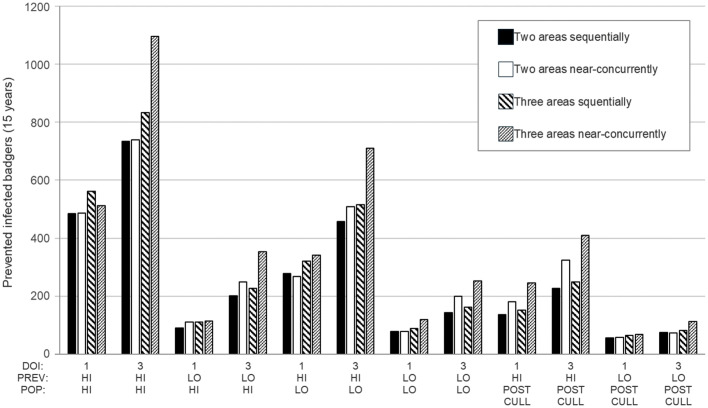
Number of infected badgers prevented across multiple areas assuming the same total human resource.

Better performance would logically be expected from treating three areas compared to two, because of the greater area of land vaccinated. However, near concurrent treatment of two areas was often better than sequential treatment of three as time between vaccinations was shorter (Fig 4). Equal or additional benefit was nearly always seen with concurrent vaccination of two areas using V12 and three using V13 strategies compared to treating two or three areas sequentially with V11 strategy. Only the combination V13, DOI1, POP HI and PREV HI resulted in a benefit from vaccinating three areas sequentially, when V11 prevented 50 more infected badgers than V13 (562 and 512, respectively). There was minor benefit from a sequential strategy in two other cases, but the difference was no more than ten infected badgers. In every case, the benefit of near-concurrent vaccination was greater under DOI3 than DOI1, as untreated areas continued to receive protection from persisting immunity during non-vaccination years. Under the near-concurrent V12 and V13 strategies, approximately 30% of badgers were immune for at least 12 of the 15 year vaccination programme in all three areas, whereas under the sequential V11 strategy, the areas were without protection for one third for DOI1or half the time for DOI3 (Fig 3).

### Disease eradication

**Fig 5:**
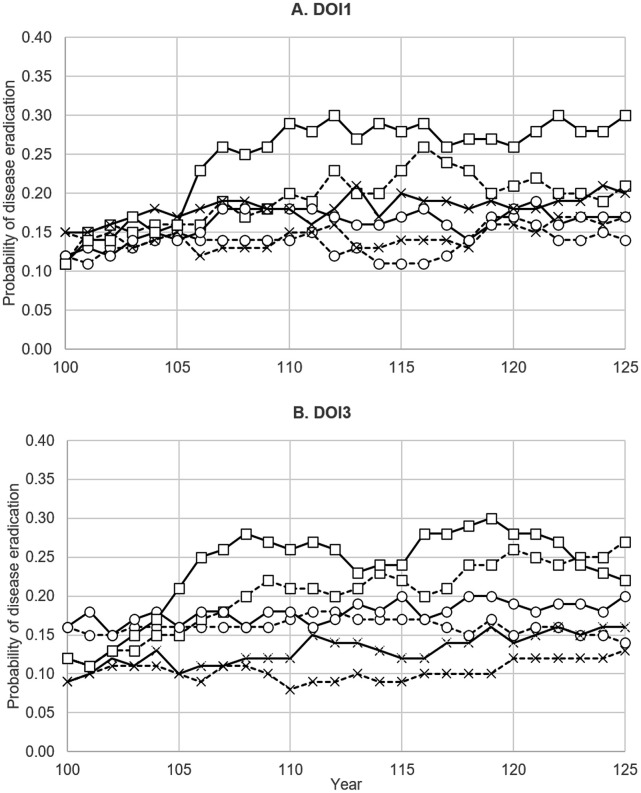
Probability of disease eradication (zero infected badgers in the core area). V11 solid line, BAU dashed line. POP HI circles, POP LO cross, POP POST CULL squares.

Choice of strategy may be influenced by the probability of disease eradication (here defined as zero infected badgers within the core area). It can be seen from Fig 1 that the average number of infected badgers present in the arena across all simulations under every combination of conditions was always more than zero using these time-limited strategies. Even under the most beneficial conditions of TRAP HI, POP POST CULL and PREV LO, the probability of achieving eradication was never more than 0.32 and duration of immunity made little difference to eradication probability in this scenario as vaccination had little effect on the level of infection present (Fig 5). Under PREV HI conditions, probability of disease eradication never exceeded zero.

## Discussion

Operational managers will want to know the likely effectiveness of vaccinating a target population, so factors used in this study were parameterised to encompass the full range of values seen in the field when possible. We have shown that among the most influential factors are those that can be estimated by field survey, namely population density (group size) and background disease prevalence. Our modelling suggests that vaccination can substantially reduce the number of infected badgers present in a 100 km^2^ area when population density (group size) and background disease prevalence are high. When density and disease prevalence are low, we show that vaccination can reduce infection by a similar proportion but less in terms of absolute numbers, and when a population that has a naturally low prevalence has recently been culled, very little additional reduction in infection is seen from vaccination, although in a post-cull scenario vaccination can be particularly effective [31]. Areas of differing population density and disease prevalence can therefore be selected with the aim of achieving either the largest reduction or lowest total number of infected animals. However, successful eradication of disease is unlikely with the strategies explored here, even where population density and disease prevalence are low. This is in line with earlier modelling studies of pure vaccination strategies where vaccination would need to proceed for much longer than simulated here [22,33]. Managers should therefore take steps to discover the basic demographic and epidemiological parameters of target populations, if at all possible. These data should preferably be obtained by systematic survey and disease testing of the badger population, as data on disease in cattle is not always a reliable predictor of disease in badgers, since most cattle infection is caused by cattle to cattle spread [1].

However, duration of immunity, which is highly influential on the success of vaccination, remains uncertain and overly optimistic assumptions about the true value should be avoided when estimating vaccination success. Duration of immunity determines the proportion of animals with ongoing protection from disease and is therefore especially important for infrequent vaccination strategies. In some cases (HIGH POP, HI PREV, V13), DOI3 resulted in twice as many prevented infected badgers over 15 years as DOI1. Trapping efficiency also affects success, but it is difficult to increase without significant additional resource; the likely achievable trapping rate should be taken into account when estimating treatment outcomes.

The choice of strategy is where managers can have the greatest influence on success and value for money in the deployment of resources. In a single area, a better outcome was usually seen from a short-term, frequent application strategy. A five year V11 strategy usually secured the greatest benefit in terms of disease reduction over fifteen years compared to V12 or V13, especially when population density and disease prevalence were high, and always reduced disease the quickest. However, adopting a longer, less frequent V12 or V13 strategy allows multiple areas to be treated near-concurrently by a single vaccination team. While substantial additional benefit from treating multiple areas concurrently was only seen if the true value of DoI is more than one year, it was usually the best approach and was rarely less effective than sequential strategies. The potential benefit offered from more efficient deployment of field resources, especially when areas are located close to one another may be enough for a concurrent strategy to be preferred in most cases.

### Model limitations

In this study, the model was extended to simulate populations with different population density and disease prevalence to Woodchester Park, where empirical parameter data was originally collected. At the time, Woodchester Park had a large mean group size (6.3) and high disease prevalence (0.17), but we have successfully validated the model at a smaller group size (4.5) in a study of a TVR trial in Northern Ireland [23], so modifying the model to operate around this range is reasonable. However, there is limited data available for stable, low disease prevalence badger populations or for the rate of disease prevalence return in culled populations, so results from these parts of the study have greater uncertainty. The removal of badgers during the Randomised Badger Control Trial (RBCT) [34] successfully reduced disease in cattle, but it returned to near-pre control levels within about five years of the end of culling, very much faster than the rate of return after culling seen in the badger population in our modelling. While the quick return post-RBCT may have been caused by factors other than badgers, it is likely our model is predicting a disease return rate that is too slow.

As we used social group size rather than group density to modify population density, disease transmission in low density populations may not be as well represented. In particular, the ratio of within- to between-group transmission probability is based on the configuration of groups at Woodchester Park which may not accurately reflect the ratio in low density areas, which may have an unknown effect on the modelled representation of disease. This may not be critical as long as the assumption of contiguous territories remains true and normal social processes are maintained, as we adjust transmission probability to achieve the desired disease prevalence value.

Model output was included for up to 25 years after the start of vaccination, at which point there is considerable inherent uncertainty in the output. Changes in government policy or badger populations, or cattle vaccination over time will add to this uncertainty.

## Conclusion

A large-scale wildlife management intervention is expensive and complex, and outcomes are highly uncertain, so we used an existing simulation model of badgers and bTB to estimate the effectiveness of different badger vaccination strategies under different field, operational and biological conditions. Intermittent vaccination, splitting effort between two areas for example, permits a much larger area to be vaccinated for the same cost, and is not detrimental. It is hoped that operational managers can use this information to prioritise areas for treatment, determine the best strategy and estimate likely success, or at least understand the potential best- and worst-case outcomes, given local conditions. This understanding will be improved if empirical data is known for the target badger population. Near concurrent treatment of multiple areas is possible where vaccination is not applied every year and may offer advantages in terms of operational efficiency and disease outcomes. We show that duration of vaccine-induced immunity has a strong impact on the success of vaccination, and further work to understand this would aid in the choice of strategy.

This result of intermittent management may be of general applicability for wildlife disease, and should be investigated for other geographic locations, and other diseases.

## Supporting information

S1 File Model ODD protocol. (DOCX)

S2 File Additional scenarios. (DOCX)

S3 File Data for graphs. (XLSX)
