# Supplementary material for: A simulation model to predict the most efficient way to utilise operational resources when vaccinating badgers against bTB
Source: PLoS One. 2026 Jul 27;21(7):e0354329. doi: 10.1371/journal.pone.0354329 (PMC13405079; doi:10.1371/journal.pone.0354329)
Supplement: S2 File — (DOCX) [file pone.0354329.s002.docx]

**Supplementary Information S2. Additional scenarios: the effect of variation across the full range of each factor.**

Figures show output for factor values not included in the main text, namely PREV MED, DOI2, DOI LIFE, TRAP LO and TRAP HI. To avoid an excessive number of figures, (there are 432 combinations across the full range of all parameters), we isolate the effect of each factor on the efficacy of vaccination as expressed by the number of infected badgers by showing the effect of varying each parameter while maintaining others at a constant value.

| *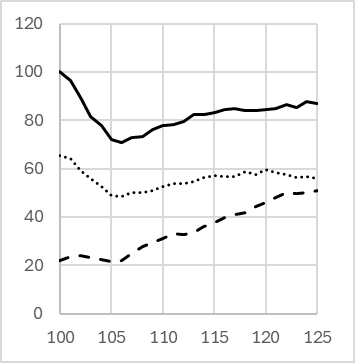* | *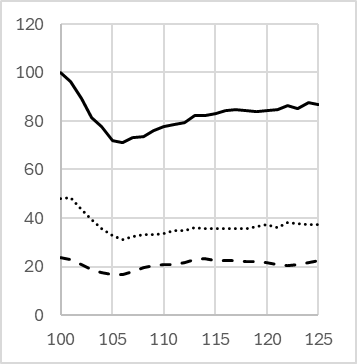* |
| --- | --- |
| *A. Population density. POP HI solid line, POP LO dotted line, POP POST CULL dashed line. Other factors: PREV HI, DOI1, TRAP MED* | *B. Disease prevalence. PREV HI solid line, PREV MED dotted line, PREV LO dashed line. Other factors: POP HI, DOI1, TRAP MED* |
| *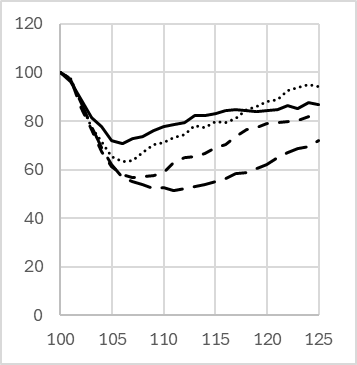* | *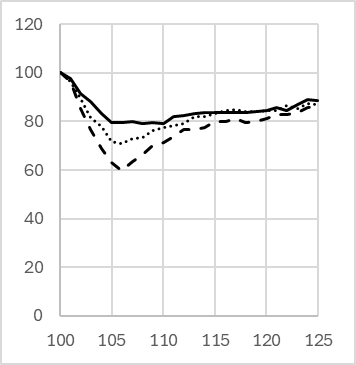* |
| *C. Duration of immunity. DOI1 solid line, DIO2 dotted line, DOI3 dashed line, DOI LIFE long dashed line. Other factors: POP HI, PREV HI, TRAP MED* | *D. Trapping efficiency. TRAP LO solid line, TRAP MED dotted line, TRAP HI dashed line. Other factors: POP HI, PREV HI, DOI1* |

**Fig 1. Effect of varying population density (group size), disease prevalence, duration of immunity and trapping efficacy under constant conditions**

The factors shown do not exhibit complex interactions so it is possible to infer the outcome for combinations not shown.

| *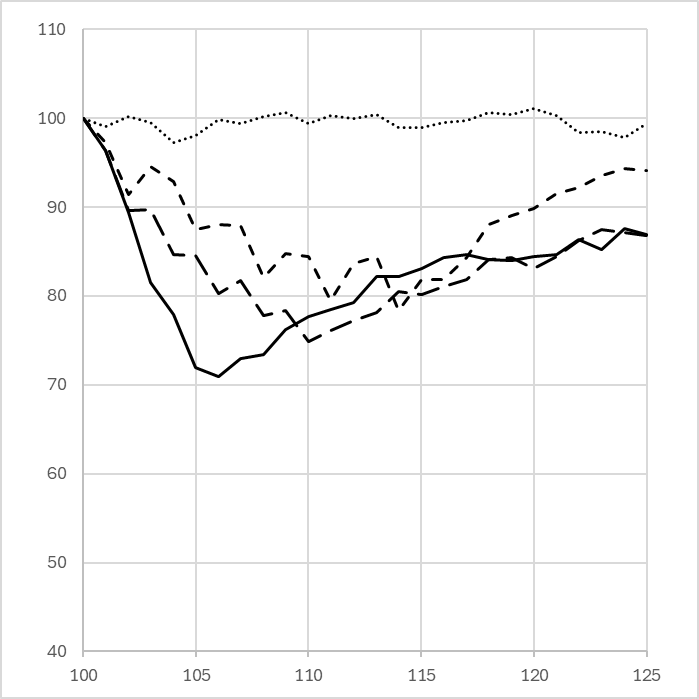* | 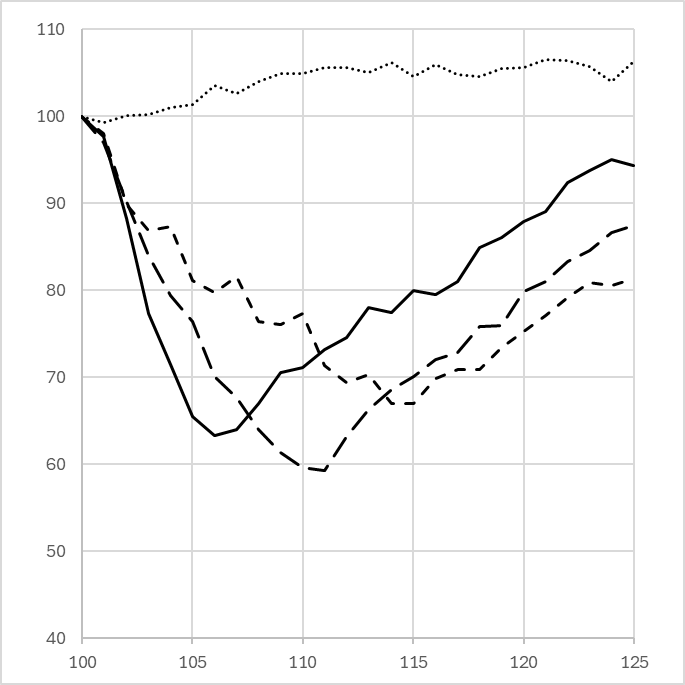 |
| --- | --- |
| *A. DOI1.* | *B. DOI2* |
| *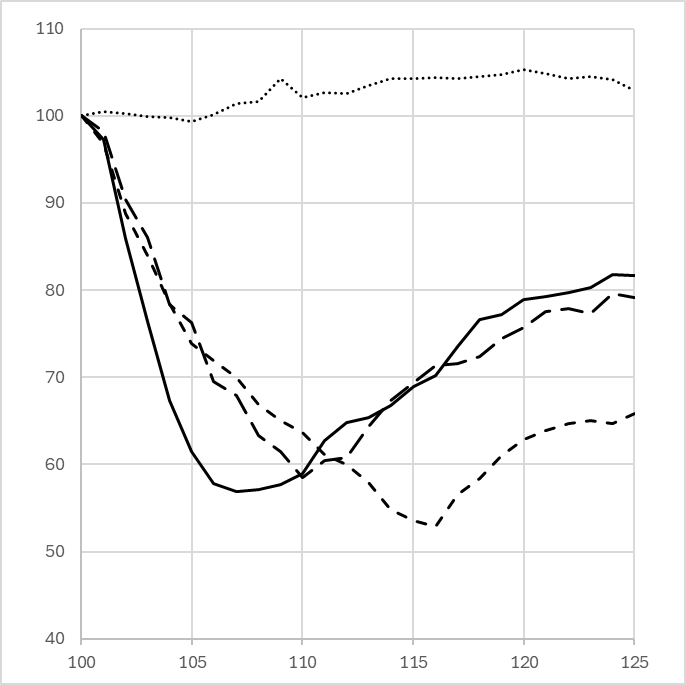* | *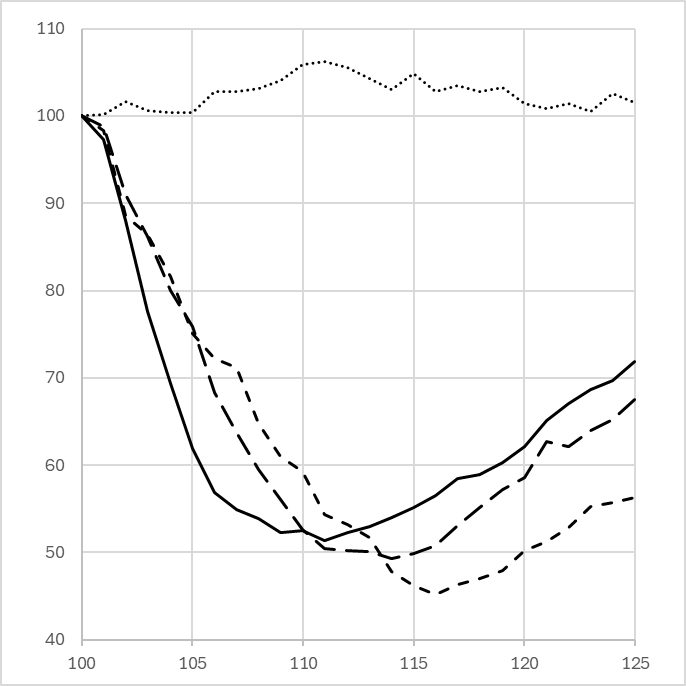* |
| *C. DOI3* | *D. DOI LIFE* |

**Fig 2. Effect of duration of immunity and vaccination strategy on the number of infected badgers.** BAU solid line, Y11 dotted line, Y12 dashed line, Y13 long dashed line. In each case, other factor values were POP HI, PREV HI, TRAP MED.

The full range of duration of immunity is shown in figure S1-2 for each of the three vaccination strategies. Other factors were held constant. Some degree of interaction is evident here, with lower frequency of vaccination showing greater efficacy at longer duration of immunity.
